# Enhancing the Localization Accuracy of UAV Images under GNSS Denial Conditions

**DOI:** 10.3390/s23249751

**Published:** 2023-12-11

**Authors:** Han Gao, Ying Yu, Xiao Huang, Liang Song, Li Li, Lei Li, Lei Zhang

**Affiliations:** 1 School of Geospatial Information, Information Engineering University, Zhengzhou 450001, China; thisxx@126.com (H.G.); neusongliang@163.com (L.S.); lili315114@163.com (L.L.); 3110100798@zju.edu.cn (L.L.); zhang295498@126.com (L.Z.); 2 31016 Troops, Beijing 100088, China; 3 61175 Troops, Nanjing 210049, China; 13224752666@163.com

**Keywords:** GNSS denial, deep learning, localization, satellite image, unmanned aerial vehicle

## Abstract

Unmanned aerial vehicles (UAVs) are widely used in many industries. The use of UAV images for surveying requires that the images contain high-precision localization information. However, the accuracy of UAV localization can be compromised in complex GNSS environments. To address this challenge, this study proposed a scheme to improve the localization accuracy of UAV sequences. The combination of traditional and deep learning methods can achieve rapid improvement of UAV image localization accuracy. Initially, individual UAV images with high similarity were selected using an image retrieval and localization method based on cosine similarity. Further, based on the relationships among UAV sequence images, short strip sequence images were selected to facilitate approximate location retrieval. Subsequently, a deep learning image registration network, combining SuperPoint and SuperGlue, was employed for high-precision feature point extraction and matching. The RANSAC algorithm was applied to eliminate mismatched points. In this way, the localization accuracy of UAV images was improved. Experimental results demonstrate that the mean errors of this approach were all within 2 pixels. Specifically, when using a satellite reference image with a resolution of 0.30 m/pixel, the mean error of the UAV ground localization method reduced to 0.356 m.

## 1. Introduction

Unmanned aerial vehicles demonstrate adaptability to complex terrains and operate at high speeds, enabling their successful application across diverse fields such as agriculture [1], firefighting [2,3,4], express transportation [5], field search and rescue [6,7], location tracking [8], and military operations [9]. These drones rely on high-precision localization information obtained from the Global Navigation Satellite System (GNSS). However, in challenging environments such as canyons, forests, deserts, water bodies, urban settings, and areas affected by human interference [10,11,12], airborne GNSS receivers are prone to problems such as signal attenuation, interference, and multipath effects. These issues lead to unreliable UAV localization [13,14,15]. Consequently, accurate localization data become unattainable for UAVs in these harsh conditions, resulting in UAV images that lack precise localization information. When used for surveying, UAV images should contain high-precision localization information. Therefore, in the case of GNSS denial, the UAV localization method proves inadequate for practical purposes and requires the use of external data to improve the accuracy of UAV images.

Schleiss [16] proposed a method for converting aerial images into street map-like representations using the conditional generative adversarial network (cGAN) [17] to achieve the visual self-localization of UAVs. However, this method exhibited a median localization error of 22.7 m, with the median error for the entire dataset being approximately 40 m, indicating a relatively large positioning error. Yol et al. [18] proposed a similarity function based on mutual information [19], which stitched a series of geographic images into a map as the localization reference. While effective in textured urban environments, this method demonstrated poor robustness in areas with weaker textures. Shan et al. [20] used the histogram of oriented gradient (HOG) [21] features for the registration of UAV frames with satellite images. Subsequently, particle filtering algorithms were utilized to localize UAVs. This method was not robust and required clear texture features, such as buildings and roads. Filho et al. [22] proposed a method based on feature recognition to identify landmark buildings and then determine UAV localization information. This method required pre-configured building images in the task area to achieve feature extraction and UAV image localization, which was not conducive to rapid localization of UAV images. Goforth et al. [23] trained a deep learning model based on the convolutional neural network (CNN) using existing satellite images. They passed UAV images and geographic reference satellite images through a CNN consisting of VGG16 neural network layers [24] and matched them, and an average localization error of less than 8 m was achieved. The method required pre-training of the model using similar images in the task area. Thus, the robustness was not high for other scenes. Saranya et al. [25,26] used satellite images with geographic information as the reference to determine the location of the UAV with the SURF algorithm as the key point detector and the RANSAC error optimization algorithm. This method had a certain robustness for rotating and scaling images but was not validated for cross-scale images.

The aforementioned methods were robust to rotation, scaling, and other transformations of the reference images. However, for cross-scale images, their robustness was poor, and the localization error was large, making it difficult to handle the entire process. In response to the shortcomings of the aforementioned methods in terms of poor cross-scale image adaptability, low localization accuracy, and lack of full-process solutions, this study proposed a method for improving the localization accuracy of UAV images with the assistance of satellite images. First, the UAV images without localization information were used to simulate the images captured under GNSS denial conditions. Commercial satellite images were utilized as reference images, and the strip relationship between UAV sequence images was used to assist in approximate location retrieval from UAV images. Subsequently, feature extraction, registration, and error removal were performed on satellite images with localization information to obtain corresponding relationships between images, thereby achieving high-precision localization of UAV images. This comprehensive and automated approach can significantly enhance localization accuracy, particularly in GNSS-denied environments. Experimental results validate the feasibility and effectiveness of the proposed method.

## 2. Principles and Methods

This study aimed to improve the localization accuracy of UAV images under GNSS denial conditions. To achieve this objective, the study followed a specific technical approach: first, it employed an image retrieval and localization method based on cosine similarity to identify single UAV images with high similarity. Subsequently, leveraging the inherent relationships within UAV sequence images, short strip sequence images were selected to aid in approximate location retrieval, resulting in the estimation of UAV image positions within high-precision satellite images. Subsequently, a deep learning image registration network, combining SuperPoint and SuperGlue, was utilized for precise feature point extraction and matching. Additionally, the RANSAC algorithm was applied to eliminate mismatched points. Through these methods, the localization accuracy of UAV images was significantly improved. Finally, the acquired feature points were utilized to calculate the homography matrix of the UAV and satellite images, enabling precise localization of the UAV image.

**Figure 1:**
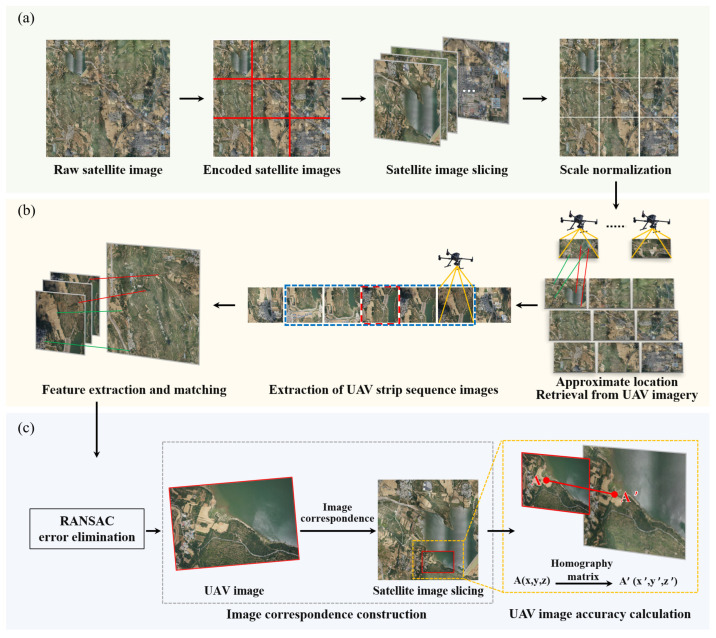
Overall design process.

The key technical flowchart of this study is shown in Figure 1. Figure 1a shows the input image and pre-processing stage. The input images consisted of original satellite images and sequence UAV images without localization information. Subsequently, the sequence UAV and satellite images underwent pre-processing, which included re-encoding, slicing, and scale normalization. Figure 1b shows the process of approximate location retrieval for UAV sequence images. The cosine similarity between the UAV image and satellite image was computed, enabling the approximate location retrieval of a single UAV image on the satellite image. Simultaneously, leveraging the strip relationship between the sequence UAV images facilitated the rapid extraction of neighboring UAV images, achieving quick approximate location retrieval and localization of UAV short strip sequence images. Figure 1c shows the accurate matching and accuracy improvement of UAV images. This study used the SuperPoint algorithm and SuperGlue neural network. Feature extraction and matching of UAV images without localization information were performed based on satellite images with localization information. Moreover, the RANSAC algorithm was used to optimize the matching results and eliminate mismatched points, leading to the derivation of the homography matrix. Consequently, the corresponding relationship between UAV images and satellite images was established. High-precision geographic coordinates could be quickly determined based on the pixel coordinates of UAV images, ultimately resulting in UAV images with high-precision localization information.

### 2.1. Approximate Location Retrieval of UAV Sequence Images

#### 2.1.1. Image Pre-Processing

To address the differences in resolution and size between satellite images and UAV images, as well as the limitations imposed by deep learning networks and computer hardware on input image sizes, this study employed a geocoding method to process cross-scale remote sensing images from various sources.

Initially, satellite images and high-resolution UAV images were geocoded based on latitude and longitude, and then, by comparing the proportion of ground objects taken by UAV images and satellite images, the scale difference proportion of the image scale can be estimated by proportion conversion and prior knowledge. According to the estimated proportion, the encoded image can be scaled, and then the image is sliced according to the coding rules. This approach effectively reduced the scale of the processed data, thereby enhancing processing efficiency. Subsequently, the segmented images were systematically renamed and arranged according to the geocoding, ensuring orderly data processing and establishing a unified coordinate system. This systematic arrangement facilitated the subsequent extraction and matching of feature points. On this basis, the UAV images were used as candidate images for sequence matching with the query images, and feature points were extracted. This method can effectively cope with the differences in resolution and size between satellite and UAV images. Further, it can adapt to the limitations of the input image size imposed by the deep learning network and performance of computer hardware. Through these steps, UAV images with high-precision localization information can be obtained. The pixel coordinates of UAV images can be utilized to quickly obtain high-precision geographic coordinates. This method not only improves the localization accuracy of UAV images but also effectively handles cross-scale remote sensing images from different sources. It demonstrated practicality and scalability in its application.

#### 2.1.2. UAV Sequence Image Extraction

A UAV captures a series of images along a specific strip during its flight, and these images exhibit strong correlations. Among these, the positional relationship between adjacent images can be determined through the relative orientation of continuous image pairs. Hence, this study utilized the strip relationships among UAV images to approximate their locations.

**Figure 2:**
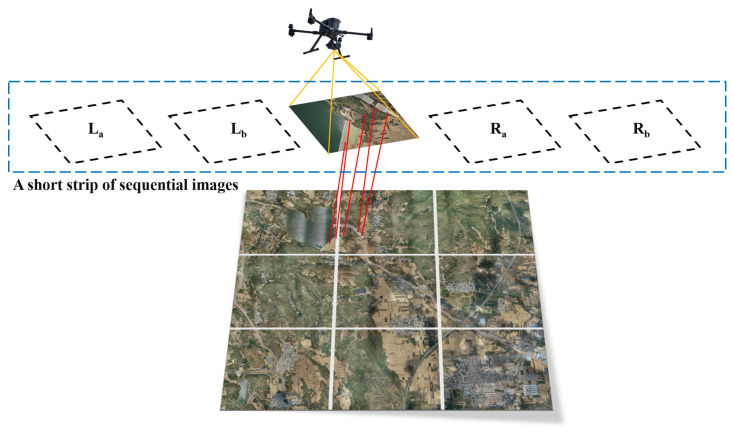
Schematic of sequence image strip extraction.

In this study, we present a novel and efficient method for UAV approximate location computation based on short strips. This method leverages the strip relationships to merge UAV images, enabling more accurate estimation of image locations and enhancing computational efficiency. Initially, this study registered the UAV sequence images with reference satellite images and calculated the cosine similarity between each pair of images. Subsequently, the UAV image with the highest cosine similarity was chosen as the center image of the strip. Additionally, the two images before and after the center image were selected, forming a sequence of five images with coarse overlapping (refer to Figure 2). Finally, the short strip was considered as a whole entity, and its approximate location was calculated separately using the satellite images.

#### 2.1.3. Calculation of Approximate Location

GNSS denial often occurs in smaller areas. Therefore, the coverage range of the satellite images selected for the experiment in this study was relatively small. In this context, this study compared UAV images with satellite images with localization information. Through meticulous feature extraction and registration processes, the study aimed to identify similarities between them. In this process, this study used a cosine similarity-based algorithm to roughly retrieve the location of the sequence UAV images on satellite images. Cosine similarity is a method of measuring the degree of similarity between two vectors in a vector space by calculating the cosine of the angle between them. If the angle between two vectors is close to 0°, their cosine similarity is high, indicating a high similarity between the two images. On the contrary, if the angle between two vectors is close to 90°, their cosine similarity is low, indicating a low similarity between the two images. The relative position of the UAV image on the satellite image can be retrieved quickly by this method. It can avoid the limitations of deep learning networks and computer hardware performance on the size of the input image. Moreover, this method is efficient and fast due to the simplicity and high efficiency of the cosine similarity computation. Additionally, its lightweight deployment eases the burden on hardware, rendering it highly practical and scalable.

**Figure 3:**
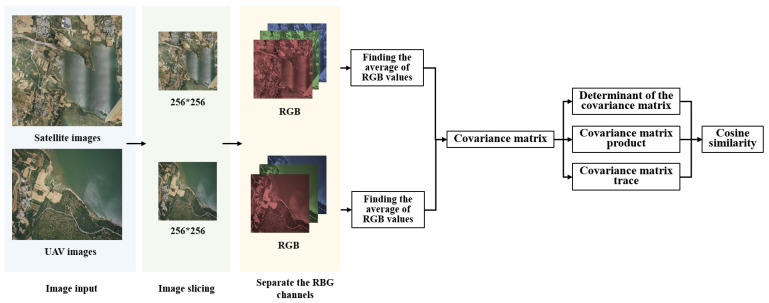
Schematic for calculating the approximate location of a UAV.

Notably, this study conducted comparative experiments on various traditional image retrieval methods, such as average hashing, interpolation hashing, perception hashing, wavelet hashing, and cosine similarity detection. It was experimentally verified that the cosine similarity detection method had a good effect on the similarity detection of cross-scale remote sensing images and UAV images from different sources. Therefore, during image pre-processing, this study used the cosine similarity detection method (refer to Figure 3). The principle is as follows:

The cosine of the angle between two vectors was utilized to measure the similarity of two images as follows:

(1) $$\cos\left(\theta \right)=\frac{A\cdot B}{∥A∥∥B∥}=\frac{\sum_{i=1}^{n}\left(x_{i}\times y_{i}\right)}{\sqrt{\sum_{i=1}^{n}{\left(x_{i}\right)}^{2}}\times \sqrt{\sum_{i=1}^{n}{\left(y_{i}\right)}^{2}}}$$

In the above equation, both *A* and *B* are eigenvectors, and *θ* is the angle between them in vector space. The cosine similarity between the reference satellite images and the UAV sequence images was calculated as a feature to evaluate the similarity between them.

### 2.2. Accurate Matching and Accuracy Improvement of UAV Images

#### 2.2.1. Feature Point Extraction Based on SuperPoint Network

**Figure 4:**
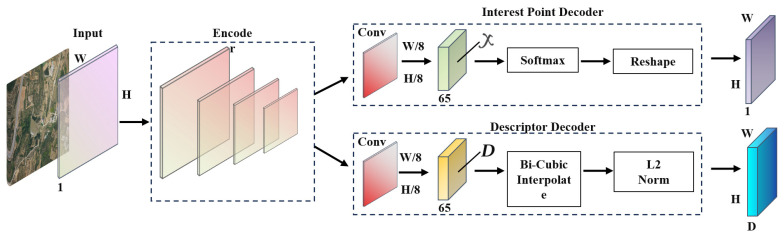
SuperPoint neural network model. Here W is the width, H is the height, *X* is the input image tensor, and *D* is the descriptor.

SuperPoint is a fully connected CNN framework [27]. The algorithm uses a lightweight convolutional neural network (CNN) to detect key points in images. The network consists of a common encoder and two decoders, which are the feature encoder, key point decoder, and descriptor decoder, as shown in Figure 4. When the algorithm processed the full-size image, it first processed and reduced the dimension of the input image through the shared feature encoder, which reduced the size of the image and obtained the image features.

Shared feature encoder. This part is constructed by the VGG modified lightweight fully convolutional neural network to encode the image. It consists of three parts: convolutional layer, Max-Pooling layer and nonlinear activation layer, there are a total of eight 33 convolutional layers, the number of convolution kernels is 64, 64, 64, 64, 128, 128, 128, 128, and the step size is 1. After each convolutional layer, the ReLU function is used, and the image size can be changed into one eighth of the output through three Max-Pooling layers.

The feature decoder. This part is divided into a feature point decoder and descriptor decoder. The feature point decoder is responsible for detecting interest points in the downscaled image, which is composed of three parts: point-by-point convolution layer, Softmax activation function, and tensor transformation function. By calculating the probability value of each pixel in the picture, it can determine whether the point is a feature point. The information contained in a pixel of the input picture to the encoder corresponds to the pixel information in the 8 × 8 area of the original image, and there are 64 channels. The encoder also sets another situation without feature points, a total of 65 channels, using multiple classifiers to remove the channels without key points, the image is changed from W/8 × H/8 × 65 to W/8 × H/8 × 64, the image is transformed from W/8 × H/8 to W × H after the reshape, and the number of channels is reduced to 1. The extracted feature points can be obtained in the output image.

The descriptor decoder part. This part consists of three parts: a convolutional layer, an interpolation function, and L2 normalization. Its role is to generate interest point descriptors for the downscaled image. It will preferentially learn the semi-dense descriptor, and then obtain the description of unit length by the bi-cubic interpolation method and L2 normalization, and then establish the loss function based on the corresponding feature points and the corresponding feature point descriptor. The algorithm then constructs the loss function based on the corresponding feature points and their descriptors. The final loss function is the sum of the losses from the feature point detector and descriptor, as shown in Equation (2).

(2) $$L(X,X^{\prime },D,D^{\prime },Y,Y^{\prime },S)=L_{p}(X,Y)+L_{p}(X^{\prime },Y^{\prime })+\lambda L_{d}(D,D^{\prime },S)$$

*L_p_* is the loss associated with the feature point, *L_d_* is the loss associated with the feature vector, X, Y, D are the feature points extracted by the convolutional network from three directions, $L_{p}\left(X,Y\right)$ and $L_{d}\left(X^{\prime },Y^{\prime }\right)$ are the loss functions of the feature point before and after the processing of the same image, respectively, *λ* is the weighting parameter, and $L_{d}\left(D,D^{\prime },S\right)$ is the loss function of the corresponding feature point descriptor.

#### 2.2.2. Feature Point Matching Based on SuperGlue Network

**Figure 5:**
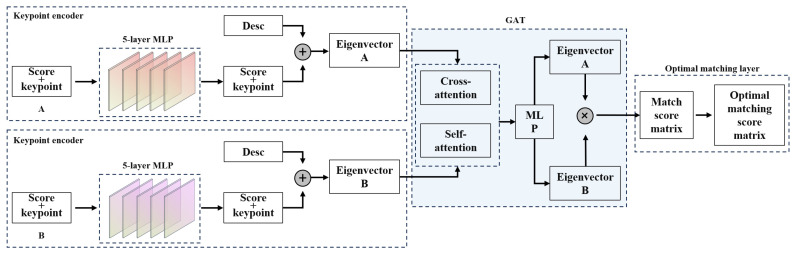
SuperGlue image-matching network.

In this study, the SuperGlue network was used to accomplish feature matching between UAV sequence images and satellite images. The SuperGlue network comprises two main modules: the attention graph neural network (GNN) and optimal matching. In image matching, learning feature matching involves finding a partial assignment between two sets of local features. This assignment problem is transformed into an optimal transportation problem through the solution of a linear assignment problem. The cost function for this optimization is predicted using a GNN. Inspired by the Transformer, it takes advantage of attention within and between images to utilize the spatial relationships of key points and their visual appearance. Consequently, the allocation structure of prediction is strengthened, allowing for effective handling of occluded and duplicate key points. The processing flowchart for the entire algorithm is generally divided into two frameworks: the attention GNN and optimal matching layer (Figure 5). The attention GNN is further segmented into the key point encoder and image attention mechanism. The key point encoder generates feature vectors, while the image attention mechanism produces the feature-matching vector. The optimal matching layer is responsible for obtaining the optimal matching score matrix and outputting the matching results.

Two modules are briefly described below. Suppose that the total number of feature points in images *A* and *B* is *M* and *N*, respectively. The position of the feature points and the description vector in image *A* are denoted as: $\left\{p_{i}{}^{A},\;i=1,\;2,\;\ldots ,\;M\right\}$ and $\left\{d_{i}{}^{A},\;i=1,\;2,\;\ldots ,\;M\right\}$; The feature point positions and description vectors in image *B* are denoted as: $\left\{p_{i}{}^{Β},\;i=1,\;2,\;\ldots ,\;M\right\}$ and $\left\{d_{i}{}^{Β},\;i=1,\;2,\;\ldots ,\;M\right\}$.

In the attention graph convolutional network, the position of the feature point is firstly increased by a feature point encoder composed of multilayer perceptron (MLP). The high-dimensional vector is obtained and added with the feature descriptor vector to obtain the initial representation of each feature point. That is:

(3) $${}^{\left(0\right)}x_{i}=d_{i}+M_{{}_{\mathrm{LP}}}(p_{i})$$

Then, a multivariate graph is constructed. The vertices of the graph are all the feature points in the two images, and the edges contain two types of edges: intra-image edges and cross-image edges. Intra-image edges connect pairs of feature points in a single image, while cross-image edges connect pairs of feature points from two images. After the graph is constructed, the features of all vertices in the graph are aggregated and updated by using the message passing mechanism. It is updated by:

(4) $${}^{\left(l+1\right)}x_{i}^{A}{=}^{\left(l\right)}x_{i}^{A}+\mathrm{MLP}(\left[{}^{\left(l\right)}x_{i}^{A}∥m_{\varepsilon \to i}\right])$$

where: [⋅||⋅] denotes join; $m_{\varepsilon \to i}$ is the information passed to node *i* from all other nodes in the graph through the self-attention mechanism. After updating by information aggregation, the description vector of each feature point is obtained as:

(5) $$f_{i}^{A}=W\cdot^{\left(L\right)}x_{i}^{A}+b,\;\forall i\in A$$

Thus, each feature vector aggregates the spatial and descriptive information of all feature points in its image and all feature points in the other image.

According to the updated features, the optimized matching layer calculates an *M* × *N* similarity matrix *S*, where each cell (*i*,*j*) in the matrix represents the similarity between the feature $f_{j}^{A}$ in image *A* and the feature $f_{j}^{B}$ in image *B*, namely:

(6) $$S_{i,j}=<f_{i}^{A},f_{j}^{B}>,\;(i,j)\in A\times B$$

Due to occlusion or a different field of view, a feature point in one image may not have a matching feature point in another image. To this end, the matrix *S* is expanded to an (*M* + 1) × (*N* + 1) matrix *S*, in which a new row and a new column are used to describe the case where the feature points do not match, namely:

(7) $${\bar{S}}_{i,N+1}={\bar{S}}_{M+1,j}={\bar{S}}_{M+1,N+1}=z\in R$$

Then, the feature-point-matching problem is transformed into an optimal transportation problem, which can be solved by the Sinkhorn algorithm. Since the Sinkhorn algorithm is derivable, it can be implemented with one network layer.

The affine transformation matrix was constructed based on the correspondence between feature-matching points of two images. This matrix was a universal linear transformation matrix that can achieve rotation, scaling, and translation transformations of images. The affine matrix can be expressed as follows:

(8) $$\left[\begin{array}{c} x_{2}\\ y_{2}\\ 1 \end{array}\right]=\left[\begin{array}{ccc} h_{11} & h_{12} & t_{x}\\ h_{21} & h_{22} & t_{y}\\ 0 & 0 & 1 \end{array}\right]\cdot \left[\begin{array}{c} x_{1}\\ y_{1}\\ 1 \end{array}\right]$$

In this equation, *h*_11_, *h*_12_, *h*_21_, *h*_22_ are the inter-image scale zoom and rotation variables, and *t_x_* and *t_y_* are the translation variables. This affine transformation matrix can be computed by importing two images matching the coordinates of the feature points (*x*_1_, *y*_1_) and (*x*_2_, *y*_2_).

Using the affine transformation matrix and mapping relationship between feature points of UAV and satellite images, the geographic coordinates of the feature points in the satellite image can be mapped to the pixel coordinates of the UAV image. This allows for the geographic coordinates of the corresponding feature points in the UAV image to be determined, thereby enhancing the accuracy of UAV ground localization.

### 2.3. Error Elimination and Optimization

During the feature matching of images from different sources, the algorithm combining SuperPoint and SuperGlue can effectively achieve image feature matching. However, differences in perspective, scale, and affine deformation exist between UAV and satellite images. Consequently, there is often a phenomenon of individual mismatched feature points in feature matching results, which affects the subsequent use of feature points as control points to correct UAV images. This study utilized the RANSAC algorithm to eliminate mismatched feature points.

The RANSAC algorithm was employed for feature point purification to enhance the estimation accuracy of the homography matrix between images, thereby improving the accuracy of image registration. It can accurately estimate model parameters even in the presence of a large number of outlier feature-matching point pairs. The inter-image homography matrix established based on the corresponding feature point relationship between two images was used as the estimation model. A homography matrix is a vector related to rotation, translation, and plane parameters [28]. The equation for the homography matrix is as follows:

(9) $$\left[\begin{array}{c} u_{1}\\ v_{1}\\ 1 \end{array}\right]=\left[\begin{array}{ccc} h_{11} & h_{12} & h_{13}\\ h_{21} & h_{22} & h_{23}\\ h_{31} & h_{32} & h_{33} \end{array}\right]\cdot \left[\begin{array}{c} u_{2}\\ v_{2}\\ 1 \end{array}\right]$$

where *u*_1_, *v*_1_, *u*_2_, *v*_2_ represent the pixel coordinates of the feature points on different images. By expanding and simplifying the matrix of Equation (10), the following equation can be obtained:

(10) $$u_{2}=\frac{h_{11}u_{1}+h_{12}v_{1}+h_{13}}{h_{31}u_{1}+h_{32}v_{1}+h_{33}}$$

(11) $$v_{2}=\frac{h_{21}u_{1}+h_{22}v_{1}+h_{23}}{h_{31}u_{1}+h_{32}v_{1}+h_{33}}$$

The feature points extracted by the SuperPoint algorithm were substituted into the model. The RANSAC algorithm randomly selected a set of feature point pairs from paired feature points as the minimum sample. The initial homography matrix model was then calculated. Subsequently, the remaining feature point data were incorporated into the model, and iterative operations were performed to verify the reliability of the feature points on the data. Generally, the errors of all matching feature point pairs were compared with a pre-set threshold. The feature points were divided into inner and outer points. Points with errors less than the threshold were regarded as inner points. During the process of obtaining the optimal homography matrix, the inner points that satisfied the geometrical constraints were retained as correctly matched feature points. Conversely, the outer points that did not meet the conditions were rejected as mismatched points. This approach effectively extracted reliable feature points and eliminated mismatched feature points, enhancing the accuracy and reliability of feature point matching.

## 3. Experimental Results and Analysis

### 3.1. Dataset and Experimental Environment

#### 3.1.1. Satellite Image Dataset

**Figure 6:**
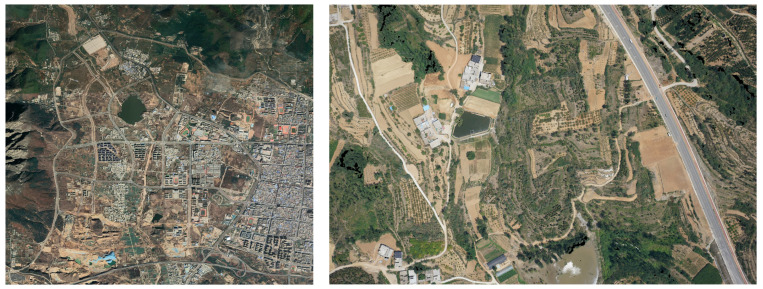
Image data of the experimental area satellite.

The satellite images employed in this paper were procured from commercial satellites. Figure 6 depicts the satellite images of the experimental area, with a magnified view of a localized region on the right. These images encompass a width of 12.79 km, a height of 12.51 km, covering an area of 159.68 square kilometers within the latitude and longitude range of 112.95–113.09° E and 34.40–34.51° N. The ground resolution stands at 0.60 m/pixel, with dimensions of 2480 × 2823 pixels. Their high ground resolutions allowed for detailed information retrieval. In the actual experimental process, these images were processed based on the specific model and efficiency requirement. To streamline data processing and enhance experimental efficiency, the input images were standardized to 1280 × 960 pixels through a normalization process.

In order to accurately simulate real-world conditions in the actual production environment, we refrained from adjusting or manipulating any image attributes, such as image quality, color scheme, brightness, white balance, contrast, and shadow, during the pre-processing of satellite images. This approach ensures the full preservation of inherent characteristics in the images. Preserving these original and crucial features is of paramount importance for subsequent tasks involving image analysis and recognition.

#### 3.1.2. Aerial Image Dataset

The UAV images used in this study were mainly taken by a DJI M300 UAV, a UAV product of DJI Innovation Technology Co., LTD., Shenzhen, China. The parameters of the equipped camera are listed in Table 1. 

**Figure 7:**
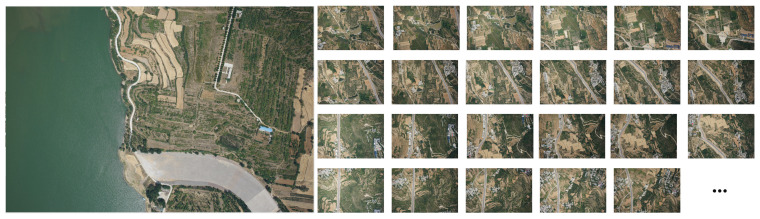
Image data of Dengfeng region in China (partial).

These images were acquired in select regions of Dengfeng and Zhengzhou, China, encompassing diverse geographical features such as cities, settlements, lakes, and vegetation. These images not only exhibited excellent representativeness but also served as a foundation for comparing and analyzing different regions. The Dengfeng area in China contributed a total of 607 UAV images, captured on 22 June 2022, with each pixel corresponding to a ground resolution of 9.91 cm (Figure 7). 

**Figure 8:**
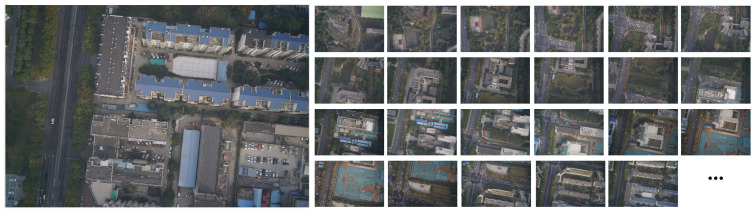
Image data of a certain region in Zhengzhou, China (partial).

Additionally, the Zhengzhou area contributed 512 UAV images, captured on 26 September 2021, with each pixel corresponding to a ground resolution of 2.94 cm (Figure 8).

In the pre-processing process of UAV images, in order to ensure the smooth development of the experiment, we only manually removed a very small number of images, which have the characteristics of large areas of cloud cover and large areas of building shadows, etc., without adjusting any attributes of the images. The original information of the image is preserved.

#### 3.1.3. Experimental Environment

The experiment was conducted using a Lenovo Y9000P laptop with an NVIDIA GeForce RTX 3060 6 GB GPU, Intel Core i7-11900H @ 2.50 GHz CPU, and 32 GB DDR4 3200 MHz RAM. The specific configuration parameters are listed in Table 2.

### 3.2. Image Approximate Location Estimation

**Figure 9:**
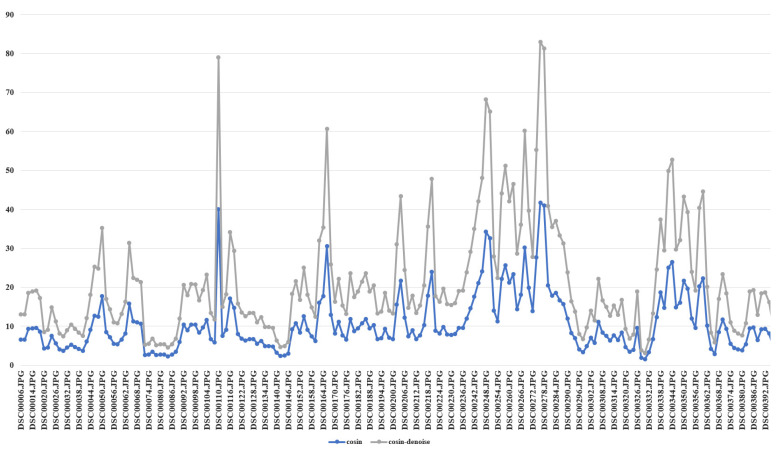
Cosine similarity matching results before and after image denoising.

Before estimating the approximate location of the image, this study conducted experiments to determine if the image needed denoising. The cosine similarity method was employed to detect the similarity between the images before and after denoising, as shown in Figure 9. The blue line represents the similarity between the images before denoising, and the gray line represents the similarity between the images after denoising. Upon analyzing the fold lines in the table, this study found that the denoising process significantly improved the overall calculation results. It effectively reduced the calculation error and facilitated a more accurate determination of the similarity threshold. Consequently, the matching results could be more precisely screened. Regarding the fitting effect, the rankings of the image retrieval estimation results remained unchanged before and after denoising. Hence, the image retrieval method proposed in this study was not sensitive to the presence of noise. Even with noise in the original image, the proposed method could accurately estimate the ranking of results.

The experimental comparisons demonstrated that utilizing cosine similarity to assess image similarity offers higher accuracy and greater robustness when handling large-format remote sensing images from diverse sources. Compared with the deep learning methods, this method did not require pre-training and supervision and had higher efficiency and faster matching speed.

**Figure 10:**
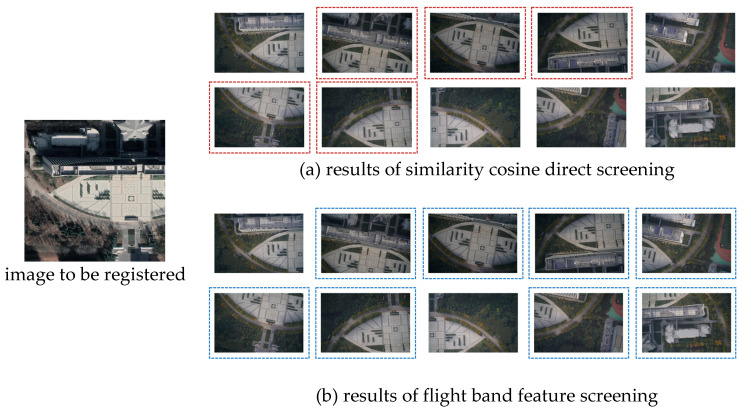
Matching results of approximate image locations.

In Figure 10, the left images represent the reference satellite images. The 10 images displayed in Figure 10a depict the sequence UAV images intended for matching. The images enclosed in red boxes represent the normally estimated images, and the UAV sequence image strip is indicated by the blue line in Figure 10b.

Upon comparing the images within the red and blue boxes, it was observed that the short strip approximate location estimation method, based on the internal relationship between UAV sequence images, effectively identifies unmatched images. Consequently, it supplements the image matching results based on cosine similarity. Moreover, extracting the sequence UAV image strip enhances the accuracy of the approximate location estimation.

### 3.3. Feature Extraction and Matching

**Figure 11:**
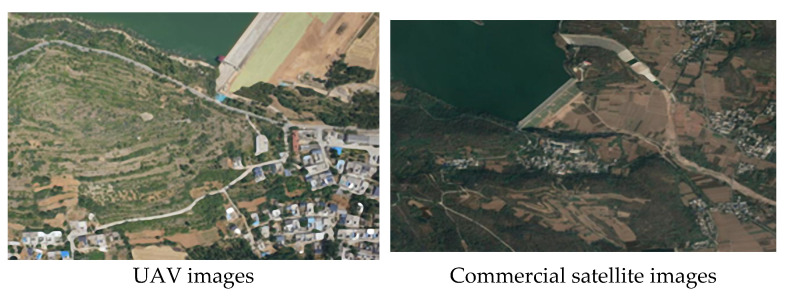

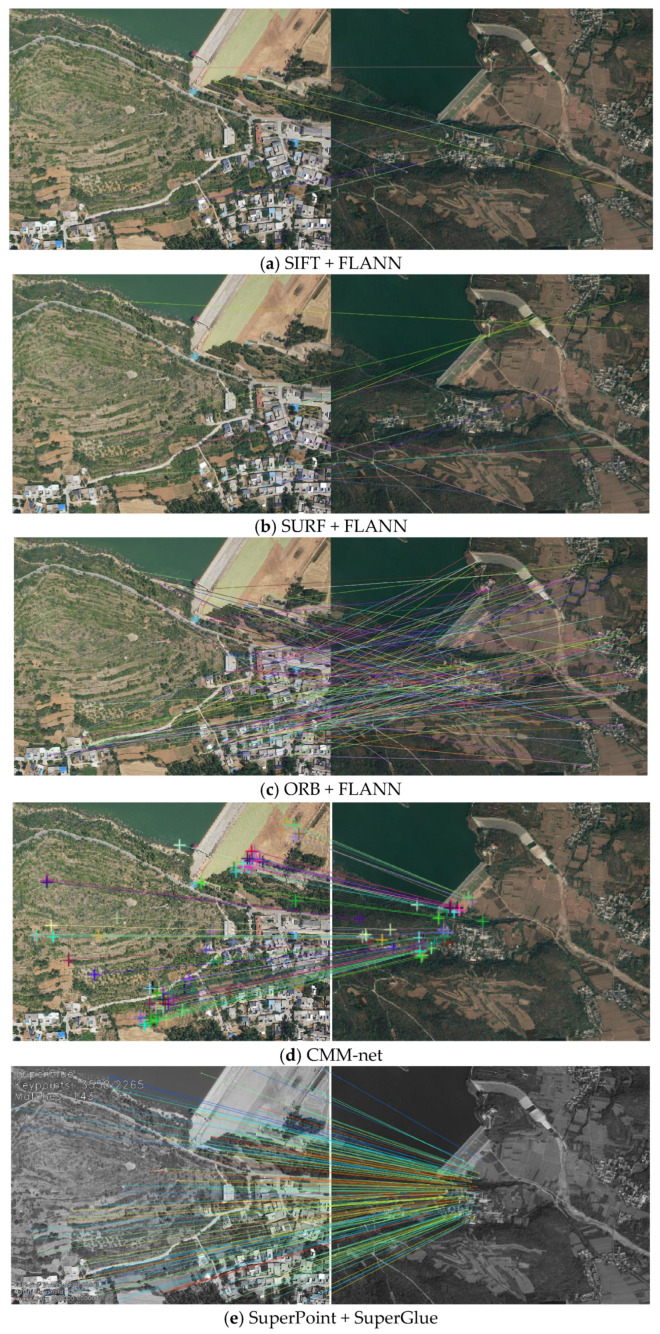
Matching results between UAV images and commercial satellite images.

In practical applications, the resolution difference between UAV and satellite images is often large, posing challenges to image matching. For better cross-scale image matching, this study adopted commercial satellite image data for the experiments (see Figure 11). Both images were sized at 1280 × 960 pixels. The left image had a resolution of 0.60 m/pixel, while the right image had a resolution of 2.39 m/pixel.

The experiment used a variety of feature-matching methods with rotation and scale invariance for comparison. These methods included traditional algorithms such as SIFT, SURF, and ORB for feature extraction, the FLANN fast nearest neighbor algorithm, the widely used deep learning CMM-Net model, and the proposed SuperPoint and SuperGlue combined network; among them, the network models used in this paper are the original default weights and hyperparameters and are not specially trained for the background of this experiment, so they are more robust and reliable.

The results are shown in Figure 11. Comparative experiments revealed that all these methods can achieve cross-scale image matching. However, traditional matching methods such as SIFT, SURF, and ORB outperformed the deep learning CMM-Net and the proposed algorithm in terms of the number of feature matching points, as demonstrated in Table 3. The traditional algorithms had a certain advantage in the number of feature points extracted, but the effect of feature matching was poorer. There were insufficient feature-matching points as well as incorrect matching. The primary reason was that the shallow feature descriptors of the neighborhood gradient of the traditional algorithms cannot adapt to the nonlinear distortion between the images from different sources. Additionally, they cannot meet the requirements of deep feature matching. This demonstrates that traditional matching algorithms cannot achieve feature matching between the UAV and satellite images.

Moreover, both the deep learning-based CMM-Net and the proposed algorithm extracted sufficient and uniformly distributed feature points in the two images, enabling efficient and accurate cross-scale image matching. CMM-Net could extract more feature points than the proposed algorithm. However, in terms of the number of successfully matched feature points, the proposed algorithm could extract more abstract feature points and construct deep-level descriptors under different lighting conditions and extreme perspective differences at varying scales. Even with a resolution difference of approximately 4 times, key points with strong robustness could still be obtained. Therefore, the proposed algorithm, combining SuperPoint and SuperGlue for feature matching of UAV images and satellite images, can provide higher accuracy in matching feature point information.

### 3.4. Number of Control Points before and after RANSAC Rejection

**Figure 12:**
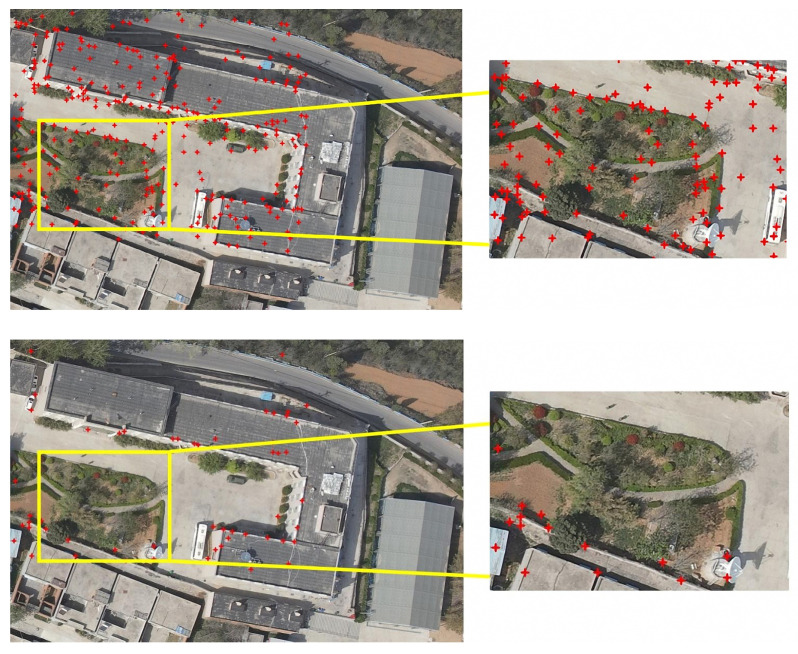
Comparison of the results of the RANSAC rejection of control point matching errors.

In the aforementioned experiment, the UAV block candidate images and satellite block query images were used as experimental data. By comparing and analyzing the feature matching results, the feature extraction results shown in Figure 12 were obtained. Numerous feature points were observed at building corners and vegetation coverage areas. Notably, the texture complexity was higher in vegetation areas. Images from diverse sources often displayed inconsistent texture features in vegetation areas due to temporal variations, posing challenges in achieving high-precision matching of feature areas at the pixel level.

To eliminate the feature-matching error points with lower accuracy, this study used the RANSAC algorithm to process the matching results. The results of removing mismatched points are shown in Figure 12. This figure shows that the feature matching accuracy in the vegetation area was low, leading to the elimination of a significant number of mismatched points. In contrast, control points were concentrated in areas with clearly defined texture information, such as building corners. By utilizing this algorithm to eliminate coarse matching feature points, high-precision feature points with robustness were extracted.

Through experimental comparisons, the matched control points were optimized for error using the RANSAC algorithm, which eliminated most of the mismatched points without evident features and retained the matched points with evident features. Consequently, the RANSAC algorithm exhibited robustness in processing this set of experimental data, ensuring accurate feature matching.

### 3.5. UAV Image Localization Accuracy Results

To validate the feasibility and accuracy of the proposed method, 12 feature points within the experimental area were chosen as checkpoints, and these checkpoints are used as the basis for accuracy evaluation. These points were then compared with the traditional methods and the localization coordinates of the UAV images, which had undergone accuracy enhancement through the proposed method. The corresponding results are listed in Table 4.

Comparative analysis revealed that using high-resolution satellite images can improve the ground localization accuracy of UAVs. The resolution of the satellite reference images used in this study can ensure the quality and accuracy of the images. Furthermore, compared with the traditional methods, the experimental results also prove that the proposed method has better accuracy and stability for the improved UAV sequence image positioning. The experimental results show that the positioning error of the proposed method is within 0.356 m, and the overall average error is within 2 pixels.

In summary, the proposed method exhibited superior accuracy and stability, significantly enhancing the ground localization precision of UAVs and yielding the expected experimental results.

## 4. Conclusions

This study aimed to improve the localization accuracy of UAV images under GNSS denial conditions. By relying on satellite reference images, the localization accuracy of UAV images was improved, and the proposed method was compared with traditional algorithms. The important conclusions of this study are as follows:

(1) This study proposed a method for approximate location retrieval of UAV sequence images based on the strip relationship and cosine similarity of UAV sequence images. Through this method, it was possible to achieve approximate location retrieval of UAV sequence images on satellite reference images. This method can quickly retrieve UAV and satellite images across scales and exhibits good robustness in different experimental areas.

(2) This study employed the SuperPoint feature extraction method based on deep learning and the feature-point-matching network, SuperGlue, which enables high-precision feature matching between UAV and satellite images. The RANSAC algorithm was used to reject mismatched feature points and select the best feature points to obtain robust high-precision control points. Experimental verification demonstrated that the proposed method significantly enhances the ground localization accuracy of UAV images. The overall scheme exhibited a mean error of less than 2 pixels, outperforming traditional feature-matching algorithms such as SIFT, SURF, and ORB.

(3) When using satellite reference images with a resolution of 0.30 m/pixel, the mean error of the UAV ground localization method was 0.356 m, further affirming the effectiveness and feasibility of the proposed method.

In summary, this study introduced a novel method for UAV ground localization, addressing the challenge of UAV image localization under GNSS denial conditions. The method yielded excellent experimental results, demonstrating high accuracy and stability, aligning perfectly with the study’s objectives.

## Figures and Tables

**Table 1:** Parameters of the camera mounted on DJI M300.

Parameter Type	Parameter Information
size	198 × 166 × 129 mm
weight	about 800 g
supported models	Matrice 300 RTK
absolute accuracy	plane accuracy: 3 cm elevation accuracy: 5 cm
minimum photo interval	0.7 s
shutter speed	mechanical shutter: 1/2000*–1 s; electronic shutter: 1/8000–1 s* aperture range: f/2.8–f/16 aperture not greater than f/5.6
ISO scope	photo: 100–25,600 video: 100–25,600

**Table 2:** Experimental environment.

Category	Configuration
model	Y9000P
graphics card	NVIDIA GeForce RTX 3060 6 GB
CPU	Intel Core i7-11900H @ 2.50 GHz
memory	32 GB DDR4 3200 MHz
operating system	Ubuntu 22.04
language environment	Python 3.6 and Python 3.9

**Table 3:** Matching results of feature points between UAV images and commercial satellite images.

Method	Image	Number of Feature Points	Number of Successful Matching Points
SIFT	left image	14,824	9
	right image	9290	
SURF	left image	**15,162**	14
	right image	8338	
ORB	left image	10,000	**176**
	right image	**9968**	
CMM-net	left image	5787	75
	right image	4388	
SuperPoint + SuperGlue	left image	3558	**143**
	right image	2265	

**Table 4:** Comparison of UAV ground localization accuracy error (m).

UAV Positioning Lifting Method	Mean Error in X Direction	Mean Error in Y Direction	Mean Error
SIFT	0.344	0.472	0.560
SURF	0.265	0.356	0.483
ORB	0.413	0.504	0.786
proposed method	0.190	0.286	0.356

## Data Availability

Data are contained within the article.
